# Demographics, Causes, and Outcome of Traumatic Brain Injury among Trauma Cases in Cameroon: A Multi-Center Five Year's Retrospective Study

**DOI:** 10.1089/neur.2022.0053

**Published:** 2022-12-26

**Authors:** Franklin Chu Buh, Germain Sotoing Taiwe, Andrew I.R. Maas, Mathieu Motah, Eric Youm, Bertrand Yuwong Wanyu, Kevin W. Wang, Peter J.A. Hutchinson, Irene Ule Ngole Sumbele

**Affiliations:** ^1^ Department of Animal Biology and Conservation, Faculty of Science, University of Buea, Buea, Cameroon.; ^2^ Panafrican Hospital Center-Douala, Buea, Cameroon.; ^3^ Department of Neurosurgery, Antwerp University Hospital and University of Antwerp, Edegem, Belgium.; ^4^ Department of Surgery, Faculty of Medicine and Pharmaceutical Sciences, University of Douala, Douala, Cameroon.; ^5^ Holo Healthcare, Nairobi, Kenya.; ^6^ Department of Emergency Medicine, University of Florida, Gainesville, Florida, USA.; ^7^ Department of Clinical Neuroscience, University of Cambridge, Cambridge, United Kingdom.

**Keywords:** Cameroon, causes, demographics, disparities in care, prevalence, traumatic brain injury

## Abstract

Traumatic brain injury (TBI) is a huge public health challenge worldwide. Epidemiological monitoring is important to inform healthcare policy. We aimed at determining the prevalence, outcome, and causes of TBI in Cameroon by conducting a 5-year retrospective study in three referral trauma centers. Data on demographics, causes, injury mechanisms, clinical aspects, and discharge status were recorded. Comparisons between two categorical variables were done using Pearson's chi-square test or Fisher's exact test. A total of 6248 cases of TBI were identified of 18,151 trauma cases, yielding a prevalence of 34%. The number of TBI cases increased across the years (915 in 2016, 1406 in 2020). Demographic data and causes of TBI were available for 6248 subjects and detailed data on clinical characteristics on 2178 subjects. Median age was 30.0 (24.0, 41.0) years. Males were more affected (80%). Road traffic incidents (RTIs; 75%) was the main cause of TBI, with professional bike riders being more affected (17%). Computed tomography (CT) imaging was performed in 67.7% of cases. Of the 597 (27.4%) cases who did not undergo neuroimaging, 311 (52.1%) did not have neuroimaging performed because of financial constraints, among which 7% were severe TBI cases. A total of 341 (19.6%) patients were discharged against medical advice, of which 83% had financial limitations. Mortality was 10.3% (225 of 2178) in the overall population, but disproportionately high in patients with severe TBI (55%) compared to those in high-income settings (27%). TBI occurrence is high in Cameroon, and RTIs are the main causes. Disparities in care provision were identified as attributable to financial constraints regarding CT scanning and continuation of care. The data presented can inform preventive interventions to improve care provision and transport policies. Implementation of a universal health insurance may be expected to improve hospital care and reduce the adverse effects of TBI among Cameroonians.

## Introduction

Traumatic brain injury (TBI) is a growing public health challenge worldwide,^1,2^ and ∼69 million persons globally sustain a TBI each year.^3^ It is a leading cause of death and disability worldwide.^4–6^ An estimated 5 million persons seek medical attention in the United States,^7^ up from 2.8 million reported by Plog and colleagues^1^ in 2015 and 2016, with ∼3.2 million persons reported as living with disability after TBI.^8–10^ TBIs are an important medical, societal, and socioeconomic problem worldwide, rendering epidemiological monitoring of incidence, prevalence, and outcome important.^2,4^ Age-standardized incidence of TBI increased globally by 3.6% between 1990 and 2016.^11^ The crude incidence rate per year in Europe ranges from 83.3 to 849 per 100,000 populations at regional levels.^12^ TBI, often referred to as a “silent epidemic,”^13^ has a lifetime prevalence of 40% in adults and is approximately 2 to 3 times more frequent in male subjects than in females.^14–16^

Although the burden of TBI is felt globally, it affects low- and middle-income countries (LMICs) disproportionately compared to high-income countries (HICs), with higher mortality rates in LMICs (18% in LMICs and 11% in HICs).^17^ In Sub-Saharan Africa (SSA), mortality attributable to TBI is high: 77% for severe TBI, 16% in moderate, and 1% in mild.^18^ The burden of TBI is high in Africa as a whole and SSA in particular, but detailed data are lacking. Currently, around one tenth of deaths in Africa are secondary to motor vehicular injury, although Africa has just 4% of the world's vehicles.^19^ Moreover, SSA is considered the global capital of road traffic deaths, most of which involve a TBI,^20,21^ and it is projected that the incidence of TBI will rise to 14 million yearly in SSA by 2050, up from the 3.2 million reported recently.^22^ This rapid increase will result in more TBIs and make SSA a focus of global attention.^23^

Cameroon, being the epicenter of SSA, is still faced with several infrastructural problems especially in terms of road infrastructure.^24^ More so, an important part of its population is young and unemployed, which exposes youths to hazardous income activities, such as professional bike riding, increasing their risk to trauma of all types including TBI. More so, poor outcome, especially in the youthful population, is likely given that they are often not able to pay for healthcare given the high cost and cannot afford private insurance services because there is not yet universal health coverage in Cameroon. Adegboyega and colleagues,^25^ in Cameroon, attempted to estimate the burden of TBI in SSA, but their review was limited to management, follow-up, and training and did not address the prevalence, incidence, causes, and outcome of TBI in SSA.

Prevalence of TBI in Sub-Saharan urban population was 19% in 2013 (841 TBIs of 4411 trauma cases),^26^ lower than the prevalence (30.9%) of TBI (415 of 1344 trauma cases) reported in a West African country.^27^ Abate and colleagues^28^ reported a prevalence of 20% in Ethiopia. These disparities in reported prevalence rates in the few studies conducted in SSA may indicate a problem of underestimation. Large, epidemiological multi-center studies considering several years are needed for a more accurate determination of the prevalence of TBI, to inform prevention and healthcare policy.

Therefore, the study aimed at determining the prevalence, main causes, and discharge status of patients with TBI in a period of 5 years from 2016 to 2020 in three level 1 trauma centers of Cameroon.

## Methods

### Study area, design, and period

We conducted a 5-year retrospective study in three referral hospitals in Cameroon. Data were collected from January 1, 2016 to December 31, 2020 in three referral hospitals of urban Yaoundé (Yaoundé Military Hospital; YMH) and Douala (Douala General Hospital [DGH] and Laquintinie Hospital of Douala [LHD])–Cameroon. These hospitals were selected because they are located in two cosmopolitan, densely populated cities of Cameroon: Douala and Yaoundé, the economic and political capitals of the country, respectively. Douala, with an estimated population of ∼3.8 million inhabitants,^29,30^ is situated on the south-eastern shore of the Wouri river estuary, on the Atlantic Ocean coast ∼230 km west of Yaoundé. It has a wet and a dry season and has temperatures ranging from 23°C to 33°C. Yaoundé, with a population of >4 million inhabitants,^31^ is situated in a hilly, forested plateau between the Nyong and Sanaga rivers in the south-central part of the country. Just like Douala, it has two seasons; a wet overcast and a dry cloudy, with temperatures varying across the year from 19°C to 31°C.^32^

These hospitals are reputed to receive most of the cases of all types of trauma given that they are among the few hospitals in the country with the required technical level needed for trauma care. In addition, they are also among the few hospitals in the country that have fully functional neurosurgical units. The DGH is a level 1 trauma center with three neurosurgeons, a multi-slice computed tomography (CT) scanner, and 0.4-Tesla (T) magnetic resonance imaging (MRI). The LHD has three neurosurgeons, a CT scanner, and 0.5-T MRI, and the YMH has two neurosurgeons, a scanner, and 0.5-T MRI.

### Study population

The population included persons of all ages who were seen with a traumatic injury who were seen within the study period, in the different health structures considered. Exclusion criteria were all non-TBIs, files lacking key information like age and sex, and obstetrical trauma. The files of patients who sustained trauma were screened and the number of cases recorded for prevalence calculations.

### Data collection

All files of patients who sustained a traumatic incident over a period of 5 years (January 1, 2016 to December 31, 2020) from the three different services of the DGH and YMH, considered to be the routine care path of TBI patients within the hospital—the emergency, reanimation, and neurosurgical services—were considered. Information concerning sociodemographic characteristics of patients (age, sex, profession, marital status, religion, and nationality) and vital signs were abstracted and recorded in the case report forms (CRFs). Hypothermia was considered to be temperatures <35°C, normal temperatures between 35°C and 37.9°C, and high temperatures or fever as from 38°C.^33,34^ Blood pressures (mm Hg) <90 mm Hg systolic or 60 mm Hg diastolic values were considered as hypotension. Systolic/diastolic values of 130–139/80–89 mm Hg were considered as stage 1 hypertension and systolic values >140 mm Hg and/or diastolic values >90 mm Hg as stage 2 hypertension.

Stage 1 and 2 hypertension were considered in this study as indicative of high blood pressure^35^; we further collected data on causes of TBI/mechanism of injury, outcome (length of stay in the hospital [LOS], discharged against medical advice [DAMA], good recovery, mental or physical disability, vegetative state [VS], or death), clinical details (medicosocial history, neurological examination, including the admission Glasgow Coma Scale [GCS], symptoms of TBI, and discharge status). Injury severity was classified according to the GCS: 3–8 for severe TBI, 9–12 for moderate, and 13–15 for mild.

At the DGH, patient files were registered by month and year; that is, January to December of each year in all three services that constitute the normal circuit of trauma patients at the DGH: emergency, surgery, and reanimation. Therefore, at a given year, we proceeded by reviewing all files monthly, from which we selected TBI cases and other traumatic cases. Trauma cases for each month were counted and recorded. Once data were extracted from TBI files to CRFs, we reclassified the files and moved to another month going from 2020 to 2017. Because of the ill-handling of files by previous research students, files of 2016 were missing at the DGH. Therefore, only registers for 2016 were considered. The same procedure was applied at the surgical unit, and care was taken not to count a file twice.

At the YMH, the same procedure was followed as at the DGH, which consisted of counting and recording the trauma cases, then counting and extracting data from TBI cases into CRFs.

Finally, at the LHD, which is noted to receive the highest number of trauma cases in the Littoral Region or Cameroon as a whole, data collection was limited to those available from the registers at the emergency department, because of the incoherent manner of data storage, and included age, sex, profession, cause and mechanism of injury, and TBI severity. As a consequence, detailed data on clinical characteristics and imaging could not be retrieved from this site. All the registers at the emergency unit between 2016 and 2020 were considered; however, the months of January to April 2016 were missing because registers could not be found.

### Discharge status and outcome

Survival status on discharge, discharge destination, if the patient was DAMA, and calculated LOS were recorded. The Glasgow Outcome Scale-Extended (GOSE) was not recorded in patient files at discharge; therefore, to record the outcome, the report at discharge on the patient's discharge files were read. The outcome on discharge was classified as death, VS, mental or physical disability, and good recovery. Good recovery was considered present if no complaints were reported at the time of discharge and all the vitals were normal. Generally, clinicians used the French jargon “RAS,” which is “Rien a signaller” meaning “Nothing to signal.”

### Statistical analysis

Required data were extracted from patient files into dedicated CRFs and checked for any errors. All forms were given unique codes and the information entered into a CSPro 7.6 data mask built by the statistician. Continuous variables are reported as medians with 25th and 75th percentiles and as means and standard deviations (SDs). Categorical variables are described as frequencies and percentages. TBI prevalence was calculated by dividing the total number of TBI cases registered within the 5-year period by the total number of trauma cases within the same period and multiplied by 100. Comparisons between two categorical variables were done using the chi-square test or Fisher's exact test, where appropriate. *p* values ˂0.05 were considered statistically significant. Analysis of data was performed using RStudio software (version 1.4.11; Posit, Boston, MA).

### Ethical clearance and administrative authorizations

Ethical clearance for the study was obtained from the institutional review board of the Faculty of Health Sciences, University of Buea (Reference No.: 1238-08). Administrative authorizations were obtained from all three referral health facilities where the study was conducted: Yaoundé Military Hospital, Douala General Hospital, and the Laquantinie Hospital of Douala. The need for informed consent was waived because this was a retrospective study on routinely collected data.

## Results

### Demographic characteristics and injury severity of traumatic brain injury cases recorded between 2016 and 2020

A total of 18,187 trauma cases were registered from 2016 to 2020, of which 36 were eliminated because they lacked essential information (sex, age). Of 18,151 cases retained, 6248 cases of TBI were recorded. The distribution of patients across the three referral hospitals was as follows: 1584 (25.3%) for the DGH; 4070 (65.1%) for the LHD; and 594 (9.5%) for the YMH. Median age of participants was 30.0 (interquartile range, 24.0, 41.0) years. The most represented age group was persons between 15 and 45 years (75%; 4689 of 6248). Males (80%; 4999) were more affected by TBI than females (20%; 1250). The majority of TBI cases were single (53%; 979 of 1847) and 39% (724 of 1847) were married. With respect to employment status, the majority of TBI cases were either professional bike riders (17%; 1041 or 6248) or unemployed (17%; 1037 or 6248), followed by students (16%; 1025) and traders (11%; 706). The majority of participants sustained a mild TBI (51.4%; 3212 of 6248), followed by 31.5% (1966) with a moderate TBI and (7.0%; 1064) with severe TBI (Table 1).

**Table 1.** Sociodemographic Characteristics of TBI Cases between 2016 and 2020

Characteristics	Overall (%)* N* = 6248	DGH (%)* N* = 1584	LHD (%)* N* = 4070	YMH (%)* N* = 594
Age, years, mean (SD)	33.3 (15.2)	32.6 (16.7)	33.5 (14.9)	33.5 (13.8)
˂15	413 (6.6%)	194 (12%)	208 (5.1%)	11 (1.9%)
15–45	4689 (75%)	1082 (68%)	3128 (77%)	479 (81%)
46–60	691 (11%)	187 (12%)	2468 (61)	367 (62)
˃60	455 (7.3%)	121 (7.6%)	297 (7.3%)	37 (6.2%)
Sex	*N* = 6247			
Male	4997 (80)	1198 (76)	3331 (82)	468 (79)
Female	1250 (20)	386 (24)	739 (18)	125 (21)
Profession	*N* = 6248			
Bike rider	1,041 (17)	180 (11)	808 (20)	53 (8.9)
Construction workers	351 (5.6)	96 (6.1)	241 (5.9)	14 (2.4)
Drivers	185 (3.0)	31 (2.0)	140 (3.4)	14 (2.4)
Employee in service	877 (14)	374 (24)	335 (8.2)	168 (28)
Health personnel	12 (0.2)	4 (0.3)	8 (0%)	0 (0%)
Manual workers	590 (9.4)	138 (8.7)	418 (10)	34 (5.7)
Security	115 (1.8)	7 (0.4)	107 (2.6)	1 (0.2)
Technicians	111 (1.8)	22 (1.4)	85 (2.1)	4 (0.7)
Traders	706 (11)	137 (8.6)	524 (13)	45 (7.6)
Others	107 (1.7)	23 (1.5)	78 (1.9)	6 (1.0)
Infants	91 (1.5)	55 (3.5)	34 (0.8)	2 (0.3)
Students	1025 (16)	298 (19)	600 (15)	127 (21)
Unemployed	1037 (17)	219 (14)	692 (17)	126 (21)
Marital status	*N* = 1846			
Single	979 (53)	631 (50)	—	348 (61)
Married	724 (39)	522 (41)	—	202 (35)
Widowed	37 (2.0)	27 (2.1)	—	10 (1.7)
Divorced	4 (0.4)	2 (0.2)	—	2 (0.2)
Not applicable	102 (5.5)	90 (7.1)	—	12 (2.1)
Missing	4402	314	4070	20
Nationality	*N* = 2427			
Cameroonian	2409 (99)	1568 (99)	247 (97)	594 (100)
Other	17 (0.7%)	10 (0.6%)	7 (2.8%)	0 (0%)
Missing	3822	6	3816	0
TBI severity	*N* = 6248			
Mild	3212 (51.4%)	922 (58.2%)	1868 (45.9%)	422 (71.0%)
Moderate	1966 (31.5%)	353 (22.3%)	1496 (36.8%)	116 (19.5%)
Severe	1064 (17.0%)	307 (19.4%)	701 (17.2%)	56 (9.4%)
Unclassified	06 (0.1%)	02 (0.1%)	05 (0.1%)	00 (0%)

DGH, Douala General Hospital; LHD, Laquintinie Hospital, Douala; YMH, Yaoundé Military Hospital.

### Prevalence of traumatic brain injury among trauma cases across the months and years between 2016 and 2020

**FIG. 1.**
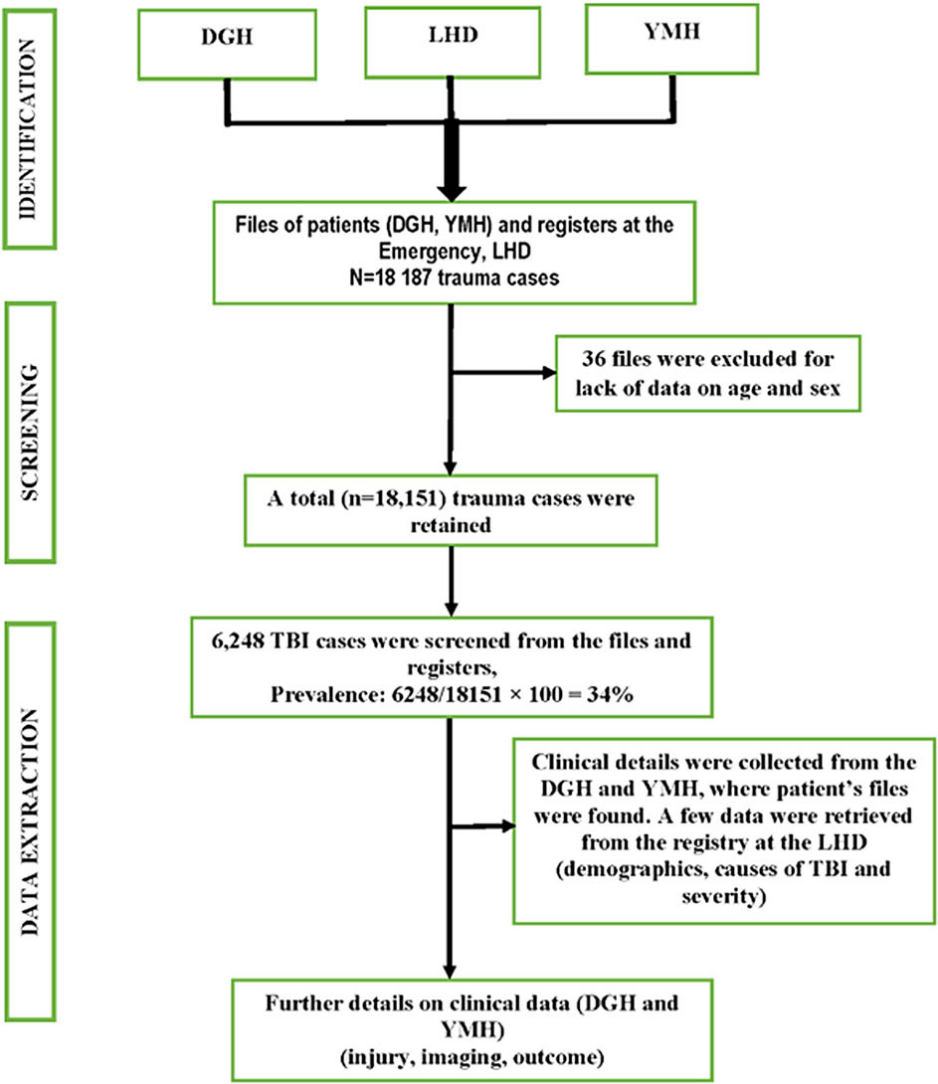
Flow chart of patients and data collection for the past 5 years (2016–2020) in Urban Douala and Yaoundé, Cameroon, June to September 2021. DGH, Douala General Hospital; LHD, Laquintinie Hospital, Douala; YMH, Yaoundé Military Hospital; TBI, traumatic brain injury.

**FIG. 2.**
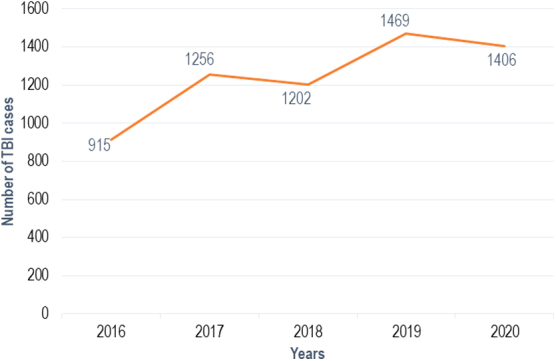
Numbers of TBI cases between 2016 and 2020, in Urban Douala and Yaoundé, Cameroon, June to September 2021. TBI, traumatic brain injury.

**FIG. 3.**
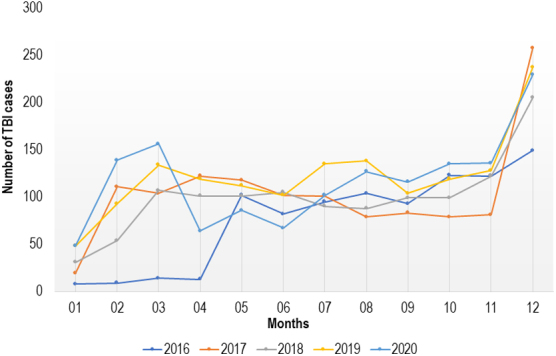
Numbers of TBI cases per month per year in all three trauma centers. TBI, traumatic brain injury.

Prevalence of TBI in the study period was 34% (Fig. 1). Between 2016 and 2020, the DHG recorded the highest TBI prevalence (37.7%), followed by the LHD (34.6%) and YMH (26.8%; Supplementary Fig. S1). Prevalence of TBI significantly increased from 31.8% in 2016 to 38.3% in 2020 (*p* ˂ 0.001; Supplementary Fig. S2). Results were consistent across centers, with the greatest increase in the YMH (Supplementary Fig. S3). Consistent with an increase in prevalence rates**, t**he number of cases of TBI also relatively increased across the years, albeit showing a slight drop in 2018 and 2020 (915 TBI cases in 2016, 1406 in 2020), as shown in Figure 2. Although some variability over the years of the study existed in the number of TBI cases across the months of the year, the highest number of TBI cases was consistently found toward the end of the year (Fig. 3).

### Clinical presentation

More detailed data on clinical and imaging characteristics were available for data collected at the DGH and YMH (2178) only. Median GCS was 14.0 (3.0–15.0). Loss of consciousness and headache were the two most common manifestations of TBI (75.3% and 39.0%, respectively; Table 2). Most TBI cases arrived at the reference hospital in less than a day (81%; 1599 of 1980), but 22 (1.7%) arrived over a month after injury. For those who arrived at the treatment center in less than a day, most (87%; 1397) arrived between ˂1 and 8 h (Supplementary Table S1). The major transportation means for the TBI cases was taxi/private vehicle (82.8%; 1639), followed by ambulances in 13.5% (267) of cases, and motorcycles were used in 36 (1.8%) cases (Supplementary Fig. S3). Most patients had stable vital signs on admission (Table 2). Median heart rate of TBI patients on arrival at the hospital was 84 (34.0, 198) beats per minute, and the majority had a normal temperature (90.0%; 1580 of 1756). Hypotension was present in 33% and in 31% of patients with moderate-to-severe TBI, respectively.

**Table 2.** Vital Signs and Clinical Symptoms of TBI Cases: Pulse, Temperature, GCS, and History of TBI, between 2016 and 2020

Characteristic: DGH and YMH	*N* = 2178
Pulse (bpm)	*N* = 1764
Median [Min, Max]	84.0 [34.0, 198]
Missing	414 (19.0%)
Blood pressure (mm Hg)	*N* = 1064
Hypotension	39 (3.7%)
Normal	309 (29.0%)
Elevated	220 (20.7%)
High blood pressure	496 (46.6%)
Missing	1114 (51.1%)
Temperature (°C)	*N* = 1756
Hypothermia	4 (0.2%)
Normal	1580 (90.0%)
Elevated	172 (9.8%)
Missing	422 (19.4%)
Respiratory rate (breathing cycle per min)	*N* = 234
Median [Min, Max]	20.0 [8, 44]
Missing	1944 (89.3%)
GCS	
Median [Min, Max]	14.0 [3.0, 15.0]
Signs and symptoms	
Loss consciousness	1639 (75.3%)
Vomiting	124 (5.7%)
Nausea	7 (0.3%)
Ear bleed	9 (0.4%)
Nasal bleed	41 (1.9%)
Headache	850 (39.0%)
Seizure	39 (1.8%)
Post-traumatic amnesia	34 (1.6%)
Agitation	145 (6.7%)
Dizziness	34 (1.6%)
Medicosocial history	*N* = 332
Diabetes	10 (3.0%)
Hypertension	75 (22.6%)
Smoking	32 (9.6%)
Alcohol	251 (75.6%)
Missing	1846 (84.8%)

DGH, Douala General Hospital; YMH, Yaoundé Military Hospital; GCS, Glasgow Coma Scale; bpm, beats per minute.

CT scanning was performed in 68% (1284 of 1896) of cases and MRI in 1% (15 of 1896). Of the 597 patients not undergoing imaging, 75% (311) did not consent to the exam because of financial constraints, among which 7% (21) had severe TBI and 23% (73) had moderate TBI. Imaging showed traumatic abnormalities in 60.3% (783) of cases. Acute subdural hemorrhage (23.1%; 181), extradural hematoma; (22.2%; 174), intracerebral hemorrhage (21.5%; 168), and cerebral contusion (20.9%, 164) were the four most common traumatic lesions registered among TBI cases (Table 3).

**Table 3.** Clinical Investigations and Type of TBI Cases between 2016 and 2020

Complementary parameters	Total (*N* = 1896)	DGH (*N* = 1305)	YMH (*N* = 591)	*p* value
CT scan	1284 (67.7%)	946 (72.5%)	338 (57.2%)	˂0.001
MRI	15 (0.8%)	15 (1.1%)	0	
No CT scan/MRI	597 (31.5%)	344 (26.4%)	253 (42.8%)	˂0.001
Missing	282 (12.9%)	279 (17.6%)	3 (0.5%)	
Reason for no exam performed	*N* = 415	*N* = 162	*N* = 253	
No finance	311 (74.9%)	139 (85.8%)	172 (68.0%)	˂0.001
Other	104 (25.1%)	23 (14.2%)	81 (32.0%)	
Missing	187 (31.3%)	182 (89.0%)		
Fracture	*N* = 600	*N* = 321	*N* = 279	
Yes	200 (33.3%)	111 (34.6%)	89 (31.9%)	0.5
No	400 (66.7%)	210 (65.4%)	190 (68.1%)	
Missing	699 (53.8), *n* = 1299	640 (66.6%), *n* = 961	59 (17.5%), *n* = 338	
If scan or MRI, traumatic brain abnormality	*N* = 1299	*N* = 960	*N* = 339	
No	516 (39.7%)	404 (42.1%)	112 (33.0)	
Yes	783 (60.3%)	556 (57.9%)	227 (70.0%)	0.003
Type of TBI	*N* = 783	—	—	
EDH	174 (22.2%)	—	—	
ASDH	181 (23.1%)	—	—	
CSDH	35 (4.5%)	—	—	
tSAH	10 (1.3%)	—	—	
Cerebral contusion	164 (20.9%)	—	—	
Cerebral edema	129 (16.5%)	—	—	
Mass effect	16 (2.0%)	—	—	
ICH	168 (21.5%)	—	—	
Other	25 (3.2%)	—	—	
Type of TBI	*N* = 1980	*N* = 1387	*N* = 593	
Blast	4 (0.2%)	3 (0.2%)	1 (0.2%)	
Blunt	1042 (52.6%)	694 (50.0%)	348 (58.7%)	
Laceration	897 (45.3%)	667 (48.1%)	230 (38.8%)	
Penetrating TBI	37 (1.9%)	23 (1.7%)	14 (2.4%)	
Missing	198 (9.1%), *n* = 2178	197 (12.4%)	1 (0.2%)	

DGH, Douala General Hospital; LHD, Laquintinie Hospital, Douala; YMH, Yaoundé Military Hospital; TBI, traumatic brain injury; CT, computed tomography; MRI, magnetic resonance imaging; EDH, extradural hemorrhage; ASDH, acute subdural hemorrhage; CSDH, chronic subdural hemorrhage; tSAH, traumatic subarachnoid hemorrhage; ICH, intracerebral hemorrhage.

### Causes and mechanism of injury among traumatic brain injury cases

The three most common causes of TBI recorded in the study were road traffic incidents (RTIs; 75% [4686]), assaults (13.2%; 825), and falls (9.3%; 581). Other causes included sports and industrial/occupational accidents (Table 4).

**Table 4.** Causes and Mechanism of TBI (2016–2020) in Urban Douala and Yaoundé, Cameroon

Characteristics	Overall* N* = 6248	DGH* N* = 1584	LHD* N* = 4070	YMH* N* = 594	*p* value
RTIs	4686 (75.0%)	1134 (71.6%)	3158 (77.6%)	393 (66.2%)	˂0.001
Falls	581 (9.3%)	225 (14.2%)	275 (6.8%)	81 (13.6%)	˂0.001
Assaults/violence	825 (13.2%)	139 (8.8%)	589 (14.5%)	97 (16.3%)	˂0.001
Industrial accident	78 (1.3%)	48 (3.0%)	31 (0.8%)	2 (0.3%)	˂0.001
Sports	19 (0.3%)	9 (0.6%)	6 (0.1%)	4 (0.7%)	0.006
Other	53 (0.8%)	25 (1.6%)	8 (0.2%)	17 (2.9%)	˂0.001
Missing	6 (0.1%)	3 (0.2%)	2 (0.0%)	0 (0%)	
Mechanism	*N* = 3536	*N* = 1389	*N* = 1556	*N* = 590	
Bicycle accident	8 (0.2%)	3 (0.2%)	2 (0.1%)	3 (0.5%)	
Motorcycle accident	2128 (60.2%)	715 (51.5%)	1153 (74.1%)	260 (44.1%)	0.3
Vehicle accident	805 (22.8%)	286 (20.6%)	391 (25.1%)	127 (21.5%)	0.4
Railway	10 (0.3%)	1 (0.1%)	8 (0.5%)	1 (0.2%)	0.9
Firearm	33 (0.9%)	19 (1.4%)	1 (0.1%)	13 (2.2%)	0.064
Blunt object	158 (4.5%)	88 (6.3%)	0 (0%)	70 (11.9%)	0.9
Knife/machete	33 (0.9%)	22 (1.6%)	0 (0%)	11 (1.9%)	0.4
Fall at same level	111 (3.1%)	64 (4.6%)	1 (0.1%)	46 (7.8%)	0.006
Fall higher level	183 (5.2%)	148 (10.7%)	0 (0%)	35 (5.9%)	0.9
Other	67 (1.9%)	43 (3.1%)	0 (0%)	24 (4.1%)	0.5
Missing	2713 (43.4%)	195 (12.3%)	2514 (61.8%)	4 (0.7%)	

DGH, Douala General Hospital; LHD, Laquintinie Hospital, Douala; YMH, Yaoundé Military Hospital.

The most common mechanism of injury involved a motorbike (60.2%; 2128), followed by motor vehicle crashes (22.8%; 805), whereas firearm and knife/machete (0.9%; 33) were the least frequent mechanisms by which TBI occurred.

Specific age or professional groups were more likely to have TBI incidence. RTIs were the most common cause in young adults ages 15–45 (77%; 3592) years. Male sex (81%) was significantly (*p* ˂ 0.001) more exposed to RTIs than females (19%). Professional bike riders and unemployed persons were the professions more likely to have an RTI (21% and 15%, respectively), as revealed in Table 5. On the other hand, falls were the most frequent cause of TBI in those under 15 years of age and in older persons (≥60). Falls were more frequent in males (68%) compared to females (32%), and students (27%) were more likely to have a TBI attributable to a fall than any other professional group (Supplementary Table S2).

**Table 5.** Causes of TBI According to Age, Sex, and Professional Groups: RTIs, in Urban Douala and Yaoundé, Cameroon

Characteristic	Other cause* N* = 1,561^1^	RTI* N* = 4,688	*p* value
Age, years			˂0.001
˂15	206 (13%)	207 (4.4%)	
15–45	1097 (70%)	3592 (77%)	
46–60	132 (8.5%)	559 (12%)	
˃60	126 (8.1%)	330 (7.0%)	
Sex			˂0.001
Female	376 (24%)	874 (19%)	
Male	1185 (76%)	3813 (81%)	
Profession			˂0.001
Bike rider	46 (2.9%)	995 (21%)	
Construction workers	108 (6.9%)	243 (5.2%)	
Drivers	22 (1.4%)	164 (3.5%)	
Employment in service	231 (15%)	646 (14%)	
Health personnel	2 (0.1%)	10 (0.2%)	
Infants	67 (4.3%)	24 (0.5%)	
Manual workers	153 (9.8%)	437 (9.3%)	
Security	42 (2.7%)	73 (1.6%)	
Students	347 (22%)	678 (14%)	
Technicians	23 (1.5%)	88 (1.9%)	
Traders	137 (8.8%)	569 (12%)	
Unemployed	348 (22%)	689 (15%)	
Other	35 (2.2%)	72 (1.5%)	

DGH, Douala General Hospital; LHD, Laquintinie Hospital, Douala; YMH, Yaoundé Military Hospital; RTIs, road traffic incidents.

Assault was significantly (*p* ˂ 0.001) associated with TBI in the 15–45 (89%) age group and the unemployed (24%; *p* ˂ 0.001) whereas no significant difference between males and females was observed (Supplementary Table S3).

### Discharge destination and status

Most patients were discharged to home (71.6%; 1559 of 2178). A total of 341 (19.6%) patients were discharged against medical advice, of whom 282 (82.7%) were because of financial limitations to continue treatment as well as the preference to follow traditional treatment in a further 11 (0.3%). Among those who left against medical advice, 11% (38 of 341) had severe TBI whereas 31% (105/341) had moderate TBI. Only 6.4% (140) were recommended rehabilitation services, such as physiotherapy, psychiatry, and psychosocial counseling. Overall mortality was 10.3% (225 of 2178), of whom 23 died on arrival and 202 in the hospital. Mortality rates were higher after severe TBI (51.5%; 187 of 363) compared to moderate (6.6%; 31 of 469) and mild TBI 0.5% (7 of 1344). Severe TBI thus accounted for 83.1% of overall mortality. Most patients discharged were considered to have good recovery (68.6%; 1187 of 1731), whereas 10.3% (178 of 1731) had physical disability, 9.2% (159 of 1731) had mental disability, and 6.5% (114 of 1731) had both (Table 6).

**Table 6.** TBI Outcome among Cases between 2016 and 2020 in the YMH and DGH, in Urban Douala and Yaoundé, Cameroon

Characteristics	Total (*N* = 2178)	DGH (*N* = 1584)	YMH (*N* = 594)	*p* value
Hospital stay	*n* = 1933	*n* = 1355	*n* = 583	**0.029**
˂1 day	462 (23.9%)	339 (25.0%)	123 (21.1%)	
1–7 days	1107 (57.3%)	760 (56.1%)	347 (59.5%)	
8–14 days	206 (10.7%)	135 (10.0%)	71 (12.2%)	
15–21 days	79 (4.1%)	54 (4.0%)	25 (4.3%)	
22–30 days	38 (1.9%)	38 (2.8%)	5 (0.9%)	
˃1 month	41 (2.1%)	29 (2.1%)	12 (2.1%)	
Missing	245 (11.2%)	229 (14.5%)	11 (1.9%)	
Discharge destination	*N* = 1753	*N* = 1209	*N* = 544	
Admission to ward	36 (2.1%)	33 (2.7%)	3 (0.6%)	**0.001**
ICU	3 (0.2%)	3 (0.2%)	0 (0%)	
Discharge to home	1559 (88.9%)	1072 (88.7%)	487 (89.5%)	
To rehabilitation	140 (8.0%)	90 (7.4%)	50 (9.2%)	
Other hospital	15 (0.9%)	11 (0.9%)	4 (0.7%)	
Missing	425 (19.5%)	375 (23.7%)	50 (8.4%)	
Left against medical advice	*N* = 1741	*N* = 1202	*N* = 539	
Yes	341 (19.6%)	184 (15.3%)	157 (29.1%)	**<0.001**
No	1400 (80.4%)	1018 (84.7%)	382 (64.3%)	
Missing	437 (20.1%)	382 (24.1%)	55 (70.9%)	
If yes, why	*n* = 341	*n* = 184	*n* = 157	**<0.001**
Lack of money: a	282 (82.7%)	140 (76.1%)	142 (90.4%)	
Other hospital; b	12 (3.5%)	4 (2.2%)	8 (5.1%)	
ab	40 (11.7%)	39 (21.2%)	1 (0.6%)	
ac: traditional treatmt	11 (0.3%)	2 (1.1%)	9 (5.7%)	
Missing	1833 (84.2%)	1399 (88.3%)	434 (73.1%)	
Discharge status	*N* = 1731	*N* = 1189	*N* = 542	
Good recovery	1187 (68.6%)	895 (75.3%)	292 (53.9%)	**0.002**
Physical disability: b	159 (9.2%)	84 (7.1%)	75 (13.8%)	**<0.001**
Mental disability: c	178 (10.3%)	84 (7.1%)	94 (17.3%)	**<0.001**
bc	114 (6.5%)	60 (5.0%)	54 (10.0%)	
VS	89 (5.1%)	64 (5.4%)	25 (4.6%)	**0.9**
Visual disability	1 (0.1%)	1 (0.1%)	0 (0%)	
Speech disability	1 (0.1%)	1 (0.1%)	0 (0%)	
bcf	1 (0.1%)	0	1 (0.2%)	
Missing	447 (20.5%)	395 (24.9%)	52 (8.8%)	
Death	225 (10.3%)	176 (11.1%)	49 (8.2%)	**0.051**

DGH, Douala General Hospital; LHD, Laquintinie Hospital, Douala; YMH, Yaoundé Military Hospital; TBI, traumatic brain injury; ab, lack of money + other hospital; ac, lack of money + traditional treatment; bc, mental + physical disability; bcf, mental + physical disability + speech disorder.

## Discussion

Our study aimed to determine the prevalence and causes of TBI in Cameroon, through a 5-year retrospective study in three referral hospitals in urban Douala and Yaoundé. Hospital files and registers provided insight on the prevalence and causes of TBI, but also highlighted the challenges involved in observational studies in low-resource settings and illustrated the impact of financial constraints on care provision.

Prevalence of TBI observed in the study (34%) is like that obtained by McKinglay and colleagues^36^ in New Zealand and Onwuchekwa and Echem^15^ in a West African country (Nigeria), who reported prevalence rates of 30% and 30.9%, respectively. However, the studies of Tesfaw and colleagues^37^ and Walle and colleagues,^38^ in Ethiopia, reported higher prevalence rates of 39.7% and 40.4%, respectively. On the contrary, other studies have reported lower prevalence rates: Abate and colleagues reported a pooled prevalence of 20% in Ethiopia.^28^ Qureshi and colleagues^26^ reported a prevalence rate of 19% for the capital city of Lilongwe in Malawi, and Webster and colleagues^39^ reported a prevalence of 24% in South Africa. The difference in prevalence rates may be explained by differences in study design and sample sizes, where cross-sectional studies on lower sample sizes and a very short duration of study ranging from 1 to 2 months were utilized. In addition, lower prevalence rates may partly be because the studies were more than a decade ago, and since then TBI incidence has increased in Africa, and differences in the population density.^29,30^ In contrast, the lower prevalence in South Africa may reflect a better infrastructural development and better road networks compared to other SSA countries.

Findings of this study demonstrate how the number of TBI cases varies considerably with the months of a year, with higher numbers of TBI cases toward the end of the year. The 5-year duration and larger sample size of this multi-center study, involving three referral hospitals with up to 18,151 trauma cases, allowed for more precise estimates and permitted the exploration of trends across the months of the years.

Observations from the study found that prevalence of TBI increased in Yaoundé and Douala-Cameroon from 2016 to 2020 by an absolute value of 6.5%. Accordingly, the numbers of TBI cases generally increased in these two cosmopolitan cities from 2016 to 2020. This corroborates reports in the literature showing an increase in global incidence of TBI with particular interest in LMICs, observing 3 times more new cases of TBI than HICs.^22,41^ The slight drop in the actual number of cases observed in 2018 and 2020 was also reflected in the total number of trauma cases and can be explained by the electoral and post-electoral atmosphere in 2018 and the Covid-19 confinement in 2020, which may have considerably reduced the movement of persons. The substantial increases in number of TBI cases across the years highlight the increasing burden of TBI in the resource-limited countries in which a majority of the population live in poverty, have no health insurance, and would not have finances to undergo adequate care after TBI.

Lack of general resources is reflected in the fact that three quarters of patients (83%) in this study used private transport to reach treatment centers, given that there are few or no emergency transport services. This greatly delays the arrival of TBI patients to the trauma centers and increases the risk for secondary insults. With emergency transport services, patients can be transferred in a more stable condition and, importantly, emergency departments can be notified so that they can prepare for their arrival, which is not the case with private transport.^42^

RTIs, assaults, and falls were the three most common causes for TBI in Cameroon, in line with reports in Tanzania,^43^ Malawi,^12^ Nigeria,^44^ Ghana,^45^ Egypt,^46^ Uganda,^47,48^ China,^49^ Angola,^50^ and Cameroon^51^ and in a multi-center study in selected African and Middle East countries.^17^ The high rate of RTIs in LMICs differs from HICs,^52–54^ where falls constitute the most common cause of TBI. This difference is likely related to better road infrastructure and stricter traffic safety regulations, which limit the incidence of RTIs, compared to African countries where poor road networks are a common reality.^62^ Moreover, most African countries, because of the lack of resources, import secondhand cars, whose failure rate is high, and this can contribute to traffic incidents. Also, the use of seatbelts and/or motorcycle helmets are not common practice, although present in the legislations of most African countries (94%).^55^ Besides these reasons, the population of Africa is fast growing alongside urbanization, which includes an expansion in motorization with little or no safety considerations in terms of road design and proper implementation of highway codes and safety practices.^55^ Factors associated with the likelihood of RTIs included being male (81%), having bike riding as a profession (21%), and unemployment (15%).

More than half of RTIs (60.2%) involved a motorcycle. However, the injury mechanism was not reported in most cases (43.3%), highlighting another big problem on effective documentation and recordkeeping faced by LMICs. The high involvement of motorcycles in RTIs could be explained by the non-implementation of safety measures by users, lack of follow-up of road safety procedures by authorities, increasing unemployment, and the younger population seeking jobs, therefore increasing hazardous jobs. Further, insecurity in the two English-speaking regions of Cameroon in 2016 (Anglophone crisis) led to a rural exodus of youths into Douala and Yaoundé, who had as their main activity commercial bike riding, did not wear helmets, seldom had a driver's license and who were hence ignorant of the highway code, and uncontrolled alcohol consumption.^56^ All these factors likely contribute to the high rate of RTIs in Cameroon and particularly to the involvement of motorcycles in most traffic incidents. Identification of the causes of RTIs with respect to age, sex, and profession could help in the development of preventive strategies based on characteristics of the population.

Although RTIs were the most common cause of TBI in the overall population, assaults were also common in the age group 15–45, but in contrast to other causes of injury, there was no difference between males and females, which is similar to reports by Kinscherf,^59^ who reported that assaults were most likely to occur among adolescents and young adults. It is also consistent with the reports by Jeanneret and Sand^60^ and the World Health Organization.^61^ Assaults were associated with unemployment, in line with the studies of Osakwe and Umoh,^62^ Outwater and colleagues^63^ in Tanzania, the Governance Social Development Humanitarian Conflict,^64^ and World Development^65^ reports, which revealed that unemployment was linked to more violence/assaults, especially in LMICs.

The length of hospital stay between 1 and 7 days in most of the cases is in line with the study of Tesfay^66^ in Ethiopia, where most TBI patients had a hospital stay between 1 and 10 days. The proportion of patients considered to have good recovery (68.6%) is in line with the findings of Landes and colleagues^67^ in Ethiopia and Eaton and colleagues in Malawi,^12^ but the proportion of patients discharged in the VS in this study was higher (4.1%) than that reported in a study in Malawi (0.8%).

Overall, we found a mortality rate of 10.3% across all severities and 51.5% in severe TBI cases. The overall mortality rate is similar to that reported by an Ethiopian emergency center,^67^ but differs from the 18% mortality reported by Clark and colleagues^17^ in a multi-national study including LMICs that considered only neurosurgical cases. Higher mortality rates (30.7%) have been reported by the main national trauma center in Tanzania,^68^ where the majority of trauma cases around the country converge. The mortality rate of severe TBI registered in this study (51.5%) is substantially higher than rates reported in HIC settings. Lu and colleagues^69^ reported a mortality rate of 27% for patients with severe TBI in 1996, and the European CENTER-TBI study, collecting data from 2014 to 2017, reported a mortality rate of 27.8% (Maas, personal communication). Mortality rates for severe TBI in Cameroon therefore appear to be double the rates in HIC settings. Likely explanations include the lack of appropriate pre-hospital and post-acute care, limited resources, and financial constraints to obtain necessary healthcare.

A relatively high number of patients (27.4%) in this study did not undergo neuroimaging, and in 75%, financial limitation was advanced as a hindrance to perform the required exams. A total of 19.6% of cases were DAMA, of whom 83% had financial limitations to continue treatment. We note that the number of cases of DAMA in LMICs like Cameroon is likely an underestimation of the true financial burden of treatment after TBI given that a large number of persons live in poverty with no health insurance. In addition, some patients receive treatment, but are not permitted to leave the hospital thereafter until they settle the hospital bills.^70^ This situation may adversely influence recovery and contribute to psychological distress in patients and their families. Further, there is a substantial risk of readmission and poor outcome among persons who leave the hospital against medical advice.^71^

Paradoxically, the negative consequences of financial constraints, leading to DAMA, remain largely unreported or non-investigated in LMICs, where the burden of TBI is most felt. Although affordable healthcare is considered a basic human right,^72^ in Cameroon over 60% of households cannot access appropriate healthcare because the cost is high.^73^ Nde and colleagues^73^ reported that ∼70% of Cameroonians spend out-of-pocket for their healthcare. To resolve this problem, the Ministry of Public Health in Cameroon started reflections in 2015 with the objective to implement a universal health coverage,^74,75^ of which the pilot phase was expected to start in 2018, but, unfortunately, is yet to be implemented.^76^ This delay renders it unlikely that the country can achieve the anticipated universal health coverage by 2035.^73^ These delays, in combination with the sociopolitical and security threats that the country faces in recent years, suggest that the suffering inflicted on the population because of the inability to afford healthcare will likely rise considerably.

This study clearly illustrates the challenges in conducting observational research in resource-limited settings. First, files on the year 2016 were missing in one of the participating hospitals (DHG), and in another (LHD), we had to restrict data extraction to registers at the emergency department, prohibiting the extraction of detailed clinical and imaging information. Further, at this site, the files of January to April 2016 could not be retrieved. Second, when extracted, the data showed many missing values for various characteristics. Good medical practice entails detailed recordkeeping that is comprehensive, timely, and adequate.^77^ The poor recordkeeping observed in Cameroon and other LMICs may be explained by the limited priority that is accorded to record management^80^ and to the fact that more efforts seem to be concentrated on financial implications, of which several researchers have reiterated that hospital recordkeeping is “more than just money” given that every component of it is to be treated with utmost care.^81^ LMICs still face many challenges with hospital recordkeeping, which could have adverse consequences and potentially reduce the value of healthcare and greatly affect the opportunities for and yield of research.^77^

Several studies indicate poor funding, inadequate computer and other information communication technology devices, poor skill in computing, harsh environmental conditions, and the lack of a preservation and conservation policy as key challenges that hinder adequate recordkeeping in developing countries.^78,79^ The difficulties linked to recordkeeping revealed in this study highlight the need for health systems in LMICs to invest resources to ensure that good recordkeeping is practiced. Electronic medical record systems practiced in developed countries are still making their way into most developing countries and are yet to make their effective entry in Cameroon.

### Strengths and limitations

This is the first study designed to retrospectively collect data on TBI on a large scale in Cameroon and SSA. A major strength of the study is the large data set collected over 5 years and included all age groups and professions. The results can inform healthcare policies to improve prevention and develop strategies to achieve best care services, aimed to improve the outcome for patients with TBI in Cameroon and the Sub-Saharan region. Despite the incomplete data retrieval as a result of the missing files, incomplete data entry and poor conservation of data leading to many missing values was identified as a limitation, probably reducing the statistical power or running the risk of biased estimates^82^; nevertheless, the data obtained are robust. Among the other limitations in the data sought were the lack of data on long-term outcome and a structured approach to record outcome on discharge. Despite these limitations, the results are relevant from a public health perspective.

## Conclusions and Implications

Prevalence of TBI in Cameroon, like other parts of Africa, is high and has increased by 6.5% from 2016 to 2020. The number of TBI cases per year in all hospitals relatively increased from 2016 to 2020. The main causes accounting for brain injury in Cameroon are RTIs, assaults, and falls. Mortality in patients with severe TBI was disproportionately high, unlike in HIC settings. We further demonstrate how financial constraints can result in disparities in care. Our experiences in data extraction illustrate problems specific to research in low-resource settings. Observations from the study would inform preventive actions, including healthcare and transport policies to reduce the incidence of TBI in Cameroon by developing and implementing adequate road safety policies and infrastructures. In addition, the implementation of a universal health insurance policy may improve hospital care and reduce the adverse effects of TBI among Cameroonians.

## Supplementary Material

Supplemental data

Supplemental data

Supplemental data

Supplemental data

Supplemental data

Supplemental data

## Abbreviations Used

CRFs: case report forms
CT: computed tomography
DAMA: discharged against medical advice
DGH: Douala General Hospital
GCS: Glasgow Coma Scale
GOSE: Glasgow Outcome Scale-Extended
HICs: high-income countries
LHD: Laquintinie Hospital, Douala
LMICs: low- and middle-income countries
LOS: length of hospital stay
MRI: magnetic resonance imaging
RTIs: road traffic incidents
SDs: standard deviations
SSA: Sub-Saharan Africa
T: Tesla
TBI: traumatic brain injury
VS: vegetative state
YMH: Yaoundé Military Hospital

## References

[1] Plog BA, Dashnaw ML, Hitomi E, et al. Biomarkers of traumatic injury are transported from brain to blood via the glymphatic system. J Neurosci 2015;35(2):518–526; doi: 10.1523/jneurosci.3742-14.2015 25589747 PMC4293408

[2] Peeters W, Brande RVD, Polinder S, et al. Epidemiology of traumatic brain injury in Europe. Acta Neurochir 2015;157:1683–1696; doi: 10.1007/s00701-015-2512-7 26269030 PMC4569652

[3] Dewan MC, Mbe AR, Gupta, S, et al. Estimating the global incidence of traumatic brain injury. J Neurosurg 2019;130:1080–1097; doi: 10.3171/2017.10.JNS17352 29701556

[4] Majdam M, Plancikova D, Brazinova A, et al. Epidemiology of traumatic brain injuries in Europe: a cross-sectional analysis. Lancet Public Health 2016;1(2):76–83; doi: 10.1016/S2468-2667(16)30017-2 29253420

[5] Wongchareon K, Thompson HJ, Mitchell PH, et al. IMPACT and CRASH prognostic models for traumatic brain injury: external validation in a South-American cohort. Inj Prev 2020;26(6):546–554; doi: 10.1136/injuryprev-2019-043466 31959626

[6] Nidhi K, Bommaraju S, Sandeep K, et al. The complexity of secondary cascade consequent to traumatic brain injury: pathobiology and potential treatments. Curr Neuropharmacol 2021;19(11):1984–2011; doi: 10.2174/1570159X19666210215123914 33588734 PMC9185786

[7] National Academies of Sciences, Engineering and Medicine. Traumatic Brain Injury: A Roadmap for Accelerating Progress. The National Academies: Washington, DC; 2022. 35533242

[8] Gardner AG, Zafonte R. Neuroepidemiology of traumatic brain injury. Handb Clin Neurol 2016;138(3):207–223; doi: 10.1016/B978-0-12-802973-2.00012-4 27637960

[9] Tóth A. Advanced Imaging in Traumatic Brain Injury. PhD thesis. Department of Neurosurgery, University of Pécs, Medical School: Pécs, Hungary; 2017. Available from: file:///C:/Users/ADMINI ∼1/AppData/Local/Temp/Toth_Arnold_angol_tezisfuzet.pdf; [Last accessed: December 9, 2022].

[10] Centers for Disease Control and Prevention. TBI: Get the Facts. 2019. Available from: file:///F:/PhD%20THESIS%20WRITEUP/TBI%20REF/TBI_%20Get%20the%20Facts%20_%20Concussion%20_%20Traumatic%20Brain%20Injury%20_%20CDC%20Injury%20Center.html [Last accessed: October 09, 2021].

[11] GBD 2016 Traumatic Brain Injury and Spinal Cord Injury Collaborators. Global, regional, and national burden of traumatic brain injury and spinal cord injury, 1990–2016: a systematic analysis for the Global Burden of Disease Study 2016. Lancet Neurol 2018;18(1):P56–P87; doi: 10.1016/S1474-4422(18)30415-0 PMC6291456 30497965

[12] Eaton J, Hanif AB, Grudziak J, et al. Epidemiology, management, and functional outcomes of traumatic brain injury in Sub-Saharan Africa. World Neurosurg 2018;108:650–655; doi: 10.1016/j.wneu.2017.09.084 28943422

[13] Traumatic brain injury: time to end the silence. Lancet Neurol 2010;9(4):331; doi: 10.1016/S1474-4422(10)70069-7 20298955

[14] Ndoumbe A, Ngoyong EPB, Simeu C, et al. Epidemiological analysis of 135 cases of severe traumatic brain injury managed at a surgical intensive care unit. Open J Modern Neurosurg 2018;8:119–131; doi: 10.4236/ojmn.2018.81010

[15] Onwuchekwa RC, Echem RC. An epidemiologic study of traumatic head injuries in the emergency department of a tertiary health institution. J Med Tropics 2018;20(1):24–29; doi: 10.4103/jomt.jomt_28_17

[16] Brazinova A, Rehorcikova V, Taylor MS, et al. Epidemiology of traumatic brain injury in Europe: a living systematic review. J Neurotrauma 2021;38(10):1411–1440; doi: 10.1089/neu.2015.4126 26537996 PMC8082737

[17] Clark D, Joannides A, Adeleye AO, et al.; Global Neurotrauma Outcomes Study collaborative. Casemix, management, and mortality of patients receiving emergency neurosurgery for traumatic brain injury in the Global Neurotrauma Outcomes Study: a prospective observational cohort study. Lancet Neurol 2022;21(5):438–449; doi: 10.1016/S1474-4422(22)00037-0 35305318

[18] Djentcheu V, Nguifo Fongang EJ, Owono Etoundi P, et al. Mortality of head injuries in Sub-Saharan African countries: the case of the university teaching hospitals of Cameroon. J Neurol Sci 2016;371:100–104; doi: 10.1016/j.jns.2016.10.016 27871428

[19] Saidi H, Mutiso BK, Ogengo J. Mortality after road traffic crashes in a system with limited trauma data capability. J Trauma Manag Outcomes 2014;8(1):4. 24524582 10.1186/1752-2897-8-4 PMC3937015

[20] World Health Organization. Global status report on road safety 2018. Available from: https://www.who.int/publications-detail-redirect/9789241565684 [Last accessed: June 4, 2022].

[21] United Nations. Road accidents in Africa among deadliest worldwide, UN official says, urging more action 2017. Available from: https://news.un.org/en/story/2017/10/569092-road-accidents-africa-among-deadliest-worldwide-un-official-says-urging-more [Last accessed: June 4, 2022].

[22] Wong JC, Linn KA, Shinohara RT, et al. Traumatic brain injury in Africa in 2050: a modeling study. Eur J Neurol 2016;23:382–386; doi: 10.1111/ene.12877 26435298

[23] Ndoumbe A, Motah M, Dah ARA, et al. Pediatric traumatic brain injury pattern at the General Hospital, Douala, Cameroon. Open J Modern Neurosurg 2019;9:49–60.

[24] African Development Bank Group. Transport Sector Support Program Phase 2. African Development Bank Group: Republic of Cameroon; 2015.

[25] Adegboyega G, Zolo Y, Sebopelo LA, et al. The burden of traumatic brain injury in Sub-Saharan Africa: a scoping review. World Neurosurg 2021:156:192–205; doi: 10.1016/j.wneu.2021.09.021 34520864

[26] Qureshi JS, Ohm R, Rajala H, et al. Head injury triage in a sub Saharan African urban population. Int J Surg 2013;11;265–269; doi: 10.1016/j.ijsu.2013.01.011 23380244

[27] Kapapa T. Traumatic Brain Injury in Africa: the Republic of Malawi as an example. COSECSA/ASEA Publication. East Central Afr J Surg 2014;19:55–58.

[28] Abate SM, Abafita BJ, Bekele T. Prevalence of traumatic brain injury among trauma patients in Ethiopia: systematic review and meta-analysis. Ann Afr Surg 2021;18(1):10–17; doi: 10.4314/AAS.V18I1.3

[29] World Population Review. Douala Population 2022. Available from: https://worldpopulationreview.com/world-cities/douala-population [Last accessed: February 11, 2022].

[30] DeLancey MW and Benneh G. Cameroon. Encyclopedia Britannica 2022. Available from: https://www.britannica.com/place/Cameroon [Last accessed: December 18, 2022].

[31] Britannica. “Yaoundé” National Capital Cameroon 2019. Available from: https://www.britannica.com/place/Yaounde. [Last accessed: December 18, 2022].

[32] World Population Review. Yaoundé Population 2022. Available from: https://worldpopulationreview.com/world-cities/yaounde-population [Last accessed: February 11, 2022].

[33] Centers for Disease Control and Prevention. Prevent Hypothermia and Frostbite 2019. Available from: https://www.cdc.gov/cpr/infographics/ast-frostbite.htm [Last accessed: March 25, 2022].

[34] Mayo Clinic. Hypothermia: An overview 2022. Available from: https://www.mayoclinic.org/diseases-conditions/hypothermia/symptoms-causes/syc-20352682 [Last accessed: March 5, 2022].

[35] National Heart, Lung and Blood Institute. Low Blood Pressure 2022. Available from: https://www.nhlbi.nih.gov/health/low-blood-pressure [Last accessed: March 25, 2022].

[36] McKinlay A, Grace RC, Horwood LJ, et al. Prevalence of traumatic brain injury among children, adolescents and young adults: prospective evidence from a birth cohort. Brain Inj 2009;22(2):175–181; doi: 10.1080/02699050801888824 18240046

[37] Tesfaw A, Eshetu M, Teshome F, et al. Prevalence of head injury among trauma patients at Debre Tabor Comprehensive Specialized Hospital, North Central Ethiopia. Open Access Surg 2021;14:47–54.

[38] Walle TB, Tiruneh BT, Bashah DT. Prevalence of head injury and associated factors among trauma patients visiting surgical emergency department of Gondar University Referral Hospital, Northwest Ethiopia 2016. A cross-sectional study. Int J Afr Nurs Sci 2016;9:57–61; doi: 10.1016/j.ijans.2018.08.002

[39] Webster J, Taylor A, Balchin R. Traumatic brain injury, the hidden pandemic: a focused response to family and patient experiences and needs. South Afr Med J 2015;105(3):195–198; doi: 10.7196/SAMJ.9014 26294826

[40] Ministére de l'Europe et des Affaires étrangéres; Malawi. Présentation du Malawi 2022. Available from: https://www.diplomatie.gouv.fr/fr/dossiers-pays/malawi/presentation-du-malawi/ [Last accessed: February 11, 2022].

[41] Iaccarino C, Carretta A, Nicolosi F, et al. Epidemiology of severe traumatic brain injury. J Neurosurg Sci 2018;62(5):535–541; doi: 10.23736/S0390-5616.18.04532-0 30182649

[42] Aljerian N, Alhaidar S, Alothman A, et al. Association between the mode of transport and in-hospital medical complications in trauma patients: findings from a level-I trauma center in Saudi Arabia. Ann Saudi Med 2018;38(1):8–14; doi: 10.5144/0256-4947.2018.8 29419523 PMC6074188

[43] Kiwango G, Msilanga D, Hocker M, et al. Epidemiology of traumatic brain injury patients at Kilimanjaro Christian Medical Centre, Moshi, Tanzania. Afr J Emerg Med 2013;3:4–10; doi: 10.1016/j.afjem.2013.08.012

[44] Oyedele EM, Andy E, Solomon GM, et al. The prevalence of traumatic head injury seen in a tertiary health facility in North-Central Nigeria. Int J Public Health Res 2015;3(4):127–129.

[45] Adam A, Alhassan A, Yabasin I. Incidence of traumatic brain injury in a Ghanaian Tertiary Hospital. J Med Biomed Sci 2016;5(2):5–12; doi: 10.4314/jmbs.v5i2.2

[46] Taha MM, Barakat MI. Demographic characteristics of traumatic brain injury in Egypt: hospital based study of 2124 patients. J Spine Neurosurg 2016;5(6); doi: 10.4172/2325-9701.1000240

[47] Mehmood A, Zia N, Hoe C, et al. Traumatic brain injury in Uganda: exploring the use of a hospital-based registry for measuring burden and outcomes. BMC Res Notes 2018;11:299, 2–8; doi: 10.1186/s13104-018-3419-1 PMC5952367 29764476

[48] Zia N, Mehmood A, Namaganda RH, et al. Causes and outcomes of traumatic brain injuries in Uganda: analysis from a pilot hospital registry. J Trauma Acute Care Surg 2019;4(1):259; doi: 10.1136/tsaco-2018-000259 PMC6407550 30899793

[49] Sun D, Jiang B, Ru X, et al. Prevalence and altered causes of traumatic brain injury in China: a nationwide survey in 2013. Neuroepidemiology 2019;54(2):106–113; doi: 10.1159/000501911 31851999

[50] De Oliveira AJM, Solari PN. First report of traumatic brain injury in Luanda, Angola. World Neurosurg 2020;143:362–364; doi: 10.1016/j.wneu.2020.07.152 32730976

[51] Motah M, Ndoumbe A, Gams MD, et al. Traumatic intracranial haemorrhage in Cameroon: clinical features, treatment options and outcome. Interdisc Neurosurg 2021;26:101346.

[52] Forslund MV, Perrin PB, Roe C. Global outcome trajectories up to 10 years after moderate to severe trumatic brain injury. Front Neurol 2019;10:219; doi: 10.3389/fneur.2019.00219 30923511 PMC6426767

[53] BrainLine. Leading Causes of Traumatic Brain Injury 2017. Available from: https://www.brainline.org/slideshow/infographic-leading-causes-traumatic-brain-injury [Last accessed: February 11, 2022].

[54] De Silva MJ, Roberts I, Perel P, et al. Patient outcome after traumatic brain injury in high-, middle- and low-income countries: analysis of data on 8927 patients in 46 countries. Int J Epidemiol 2009;38(2):452–458; doi: 10.1093/ije/dyn189 18782898

[55] Bezabeh GB, Souare M, Oumarou A. Road Safety in Africa: Assessment of Progresses and Challenges in Road Safety Management System. African Development Bank Group: Republic of Cameroon; 2013.

[56] Kouagheu J. Au Cameroun, dans la jungle des motos-taxisA Douala, sept accidents de la circulation sur dix sont causés par les « bend-skinneurs », ces jeunes chauffeurs sans le sou et, souvent, sans permis de conduire 2020. Journal Le Monde Afrique. Available from: https://www.lemonde.fr/afrique/article/2020/02/03/au-cameroun-dans-la-jungle-des-motos-taxis_6028276_3212.html [Last accessed: May 17, 2022].

[57] Haarbauer-Krupa J, Haileyesus T, Gilchrist J, et al. Fall-related traumatic brain injury in children ages 0–4 years. J Safety Res 2019;70:127–133; doi: 10.1016/J.JSR.2019.06.003 31847987 PMC6927527

[58] Centers for Disease Control and Prevention. Facts about Falls 2021. Available from: https://www.cdc.gov/falls/facts.html#:~:text=Falls%20Are%20Serious%20and%20Costly&text=Over%20800%2C000%20patients%20a%20year,head%20injury%20or%20hip%20fracture.&text=Each%20year%20at%20least%20300%2C000%20older%20people%20are%20hospitalized%20for%20hip%20fractures.&text=More%20than%2095%25%20of%20hip,8%20usually%20by%20falling%20sideways [Last accessed: March 19, 2022].

[59] Kinscherff R. Preventing Violence Among Adolescents and Young Adults 2015. Available from: Preventing Violence Among Adolescents and Young Adults (williamjames.edu) [Last accessed: March 30, 2022].

[60] Jeanneret O, Sand EA. Intentional violence among adolescents and young adults: an epidemiological perspective. World Health Stat Q 1993;46(1):34–51. Erratum in: World Health Stat Q 1993;46(3):209–210. 8237052

[61] WHO. Older adolescent (15 to 19 years) and young adult (20 to 24 years) mortality 2022. Available from: https://www.who.int/news-room/fact-sheets/detail/levels-and-trends-in-older-adolescent-(15-to-19-years)-and-young-adult-(20-to-24-years)-mortality#:~:text=To%20date%2C%20older%20adolescents%20and,of%20adolescents%20and%20young%20adults [Last accessed: March 30, 2022].

[62] Osakwe C, Umoh UE. The Military and Counterinsurgency Operations in Nigeria. In: Internal Security Management in Nigeria. (Mbachu O, Bature UM. eds.) Ministry of Defense Nigeria; Abuja, Nigeria; 2013; pp. 391–409.

[63] Outwater AH, Mgaya E, Msemo S, et al. Youth unemployment, community violence, creating opportunities in Dar es Salaam, Tanzania: a qualitative study. Tanzania J Health Res 2015;17(1):1–11; doi: 10.4314/thrb.v17i1.6

[64] Governance Social Development Humanitarian Conflict: GSDHC. Youth Unemployment and Violence: A Rapid Literature Review. University of Birmingham: Birmingham, UK; 2016.

[65] World development Report. Unemployment and participation in Violence 2011. World Development Report Background Paper 37p. Available from: https://web.worldbank.org/archive/website01306/web/pdf/wdr%20background%20paper%20-%20cramer.pdf [Last accessed: December 18, 2022].

[66] Tesfay M. Clinical profile and outcome of traumatic brain injury patients at the Emergency department of AaBET hospital, Addis Ababa, Ethiopia. Master's thesis. College of Health Science, Addis Ababa University: Ethiopia; 2020.

[67] Landes M, Venugopal R, Berman S, et al. Epidemiology, clinical characteristics and outcomes of head injured patients in an Ethiopian emergency centre. Afr J Emerg Med 2017;7(3):130–134; doi: 10.1016/j.afjem.2017.04.001 30456124 PMC6234141

[68] Boniface R, Lugazia ER, Ntungi AM, et al. Management and outcome of traumatic brain injury patients at Muhimbili Orthopaedic Institute Dar es Salaam, Tanzania. Pan Afr Med J 2017;26:1–7; doi: 10.11604/pamj.2017.26.140.10345 28533863 PMC5429442

[69] Lu J, Marmarou A, Choi S, Mortality from traumatic brain injury. Acta Neurochir Suppl 2005;95:281–285; doi: 10.1007/3-211-32318-x_58 16463866

[70] L'investigateur. Santé: les patients retenus pour non-paiement des frais médicaux, délivrés par l'Etat (Journal) 2020. Available from: https://www.linvestigateur.info/?Sante-les-patients-retenus-pour-non-paiement-des-frais-medicaux-delivres-par-l [Last accessed: May 17, 2022].

[71] Kalogriopoulos NA, Baran J, Nimunkar AJ, et al. Electronic Medical Record Systems for Developing Countries: Review. 2009 Annual International Conference of the IEEE Engineering in Medicine and Biology Society 2008: 1730-3; doi:10.1109/IEMBS.2009.5333561 19964260

[72] Marcoux J, Alkutbi M, Lamoureux J, et al. Discharge against medical advice in traumatic brain injury: follow-up and readmission rate. Can J Neurol Sci 2017;44(3):311–317; doi: 10.1017/cjn.2016.241 27226130

[73] Nde CJ, Raymond A, Saidou Y, et al. Reaching universal health coverage by 2035: is Cameroon on track? Universal J Public Health 2019;7(3):110–117; doi: 10.13189/UJPH.2019.070304

[74] Nde CJ, Raymond A, Saidou Y, et al. Progress towards universal health coverage: is Cameroon investing enough in primary care? Universal J Public Health 2019;7(4):171–178; doi: 10.13189/ujph.2019.070403

[75] Ministry of Public Health-Cameroon. National Health Development Plan: 2016-2020 2015. Ministry of Public Health. Republic of Cameroon.

[76] Business in Cameroon. Universal Health Coverage: SUCAM could launch pilot phase in H1-2022 2021. Available from: https://www.businessincameroon.com/public-management/2110-11985-universal-health-coverage-sucam-could-launch-pilot-phase-in-h1-2022 [Last accessed: February 3, 2022].

[77] Asogwa BE. The challenge of managing electronic records in developing countries: implications for records managers in sub Saharan Africa. Records Manag J 2012;22(3):198–211; doi:10.1108/09565691211283156

[78] Kang H. The prevention and handling of the missing data. Korean J Anesthesiol 2013;64(5):402–406; doi: 10.4097/kjae.2013.64.5.402 23741561 PMC3668100

[79] Pullen I, Loudon J. Improving standards in clinical record-keeping. Adv Psychiatr Treat 2006;12:280–286; doi: 10.1192/apt.12.4.280

[80] Abdulazeez YJ, Asunoa AA, Abolarinwa ST, et al. Challenges of record management in two health institutions in Lagos State, Nigeria. Int J Res Humanities Social Studies 2015;2(12):1–9.

[81] Mutshatshi TE, Mothiba TM, Mamogobo PM, et al. Record-keeping: challenges experienced by nurses in selected public hospitals. Curations 2018;41(1):1–6; doi: 10.4102/curationis.v41i1.1931 PMC6111626 30198294

[82] Saunders P. The implications of poor data recording in hospitals. Consulted online at Grant Thornton 2018. Available from: https://www.grantthornton.co.uk/insights/the-implications-of-poor-data-recording-in-hospitals/ [Last accessed: March 5,2022].

