# Supplementary figures and images for: Demographics, Causes, and Outcome of Traumatic Brain Injury among Trauma Cases in Cameroon: A Multi-Center Five Year's Retrospective Study
Source: Neurotrauma Rep. 2022 Dec 26;3(1):569–83. doi: 10.1089/neur.2022.0053 (PMC9879018; doi:10.1089/neur.2022.0053)

**Supplementary Figure S1:**

**
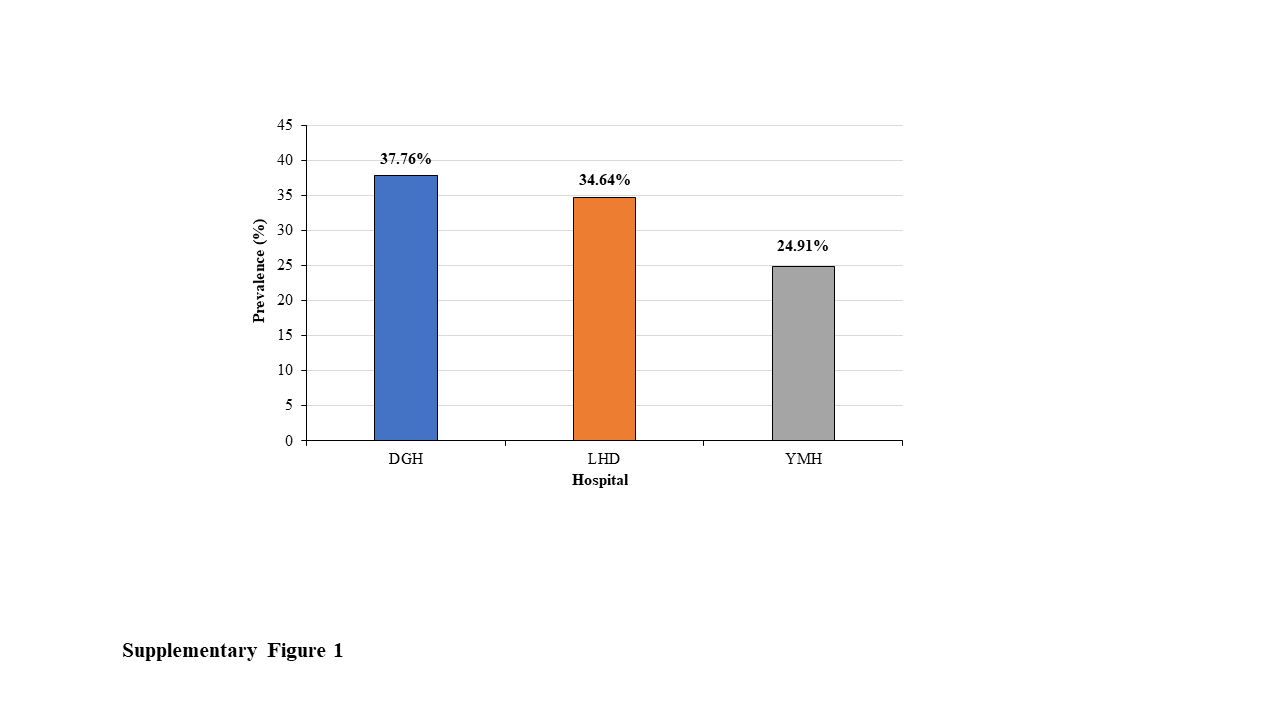
**

Supplement: Supplemental data [file Supp_FigS1.docx]

Supplementary Figure S2:


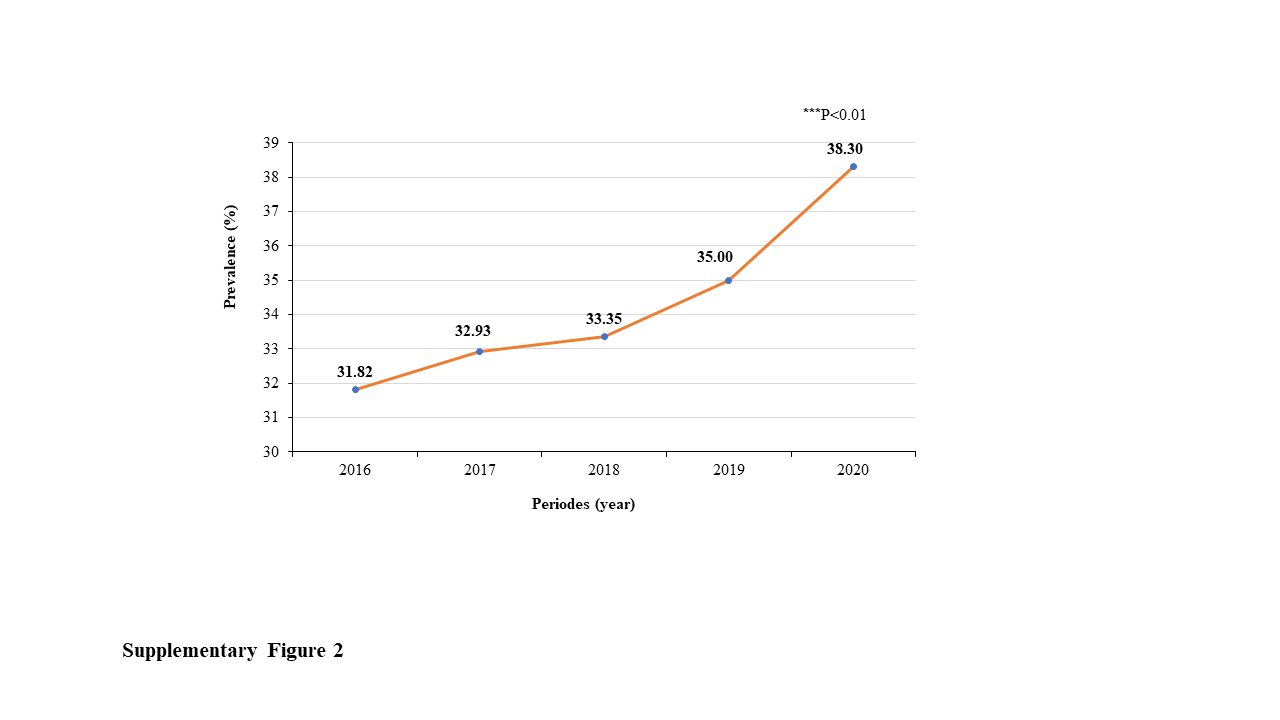

Supplement: Supplemental data [file Supp_FigS2.docx]

Supplementary Figure S3:


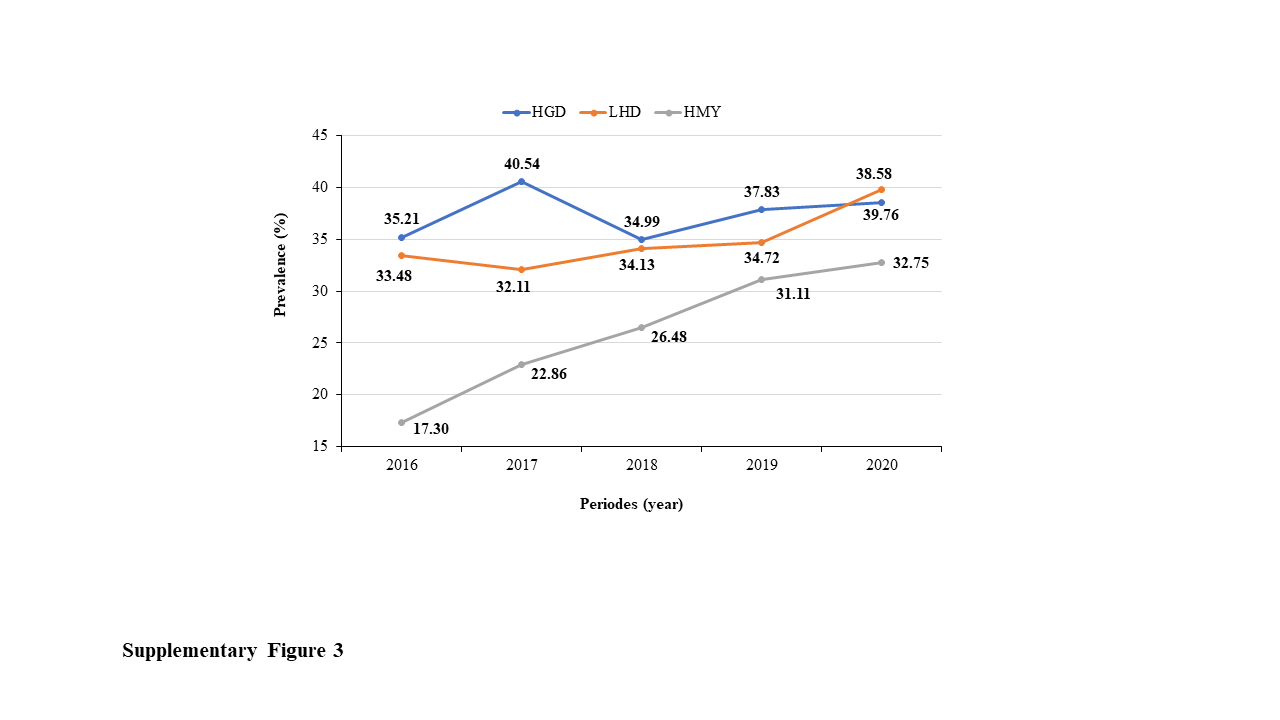

Supplement: Supplemental data [file Supp_FigS3.docx]
