# Supplementary material for: Demographics, Causes, and Outcome of Traumatic Brain Injury among Trauma Cases in Cameroon: A Multi-Center Five Year's Retrospective Study
Source: Neurotrauma Rep. 2022 Dec 26;3(1):569–83. doi: 10.1089/neur.2022.0053 (PMC9879018; doi:10.1089/neur.2022.0053)
Supplement: Supplemental data [file Supp_TableS1.docx]

**Supplementary table I: T**ime difference between injury and arrival at the treatment centres

| **Characteristics** | **Overall (%)**  **N=1980** | **DGH N=1584**  **(%)** | **YMH N=594**  **(%)** |
| --- | --- | --- | --- |
| ˂ 1 day | 1,599 (81%) | 1,174 (85%) | 425 (72%) |
| 1-7 days | 313 (16%) | 183 (13%) | 130 (22%) |
| 8- 14 days | 27 (1.4%) | 13 (0.9%) | 14 (2.4%) |
| 15-21 days | 5 (0.3%) | 1 (<0.1%) | 4 (0.7%) |
| 22-30 days | 14 (0.7%) | 6 (0.4%) | 8 (1.3%) |
| > 1 month | 22 (1.1%) | 10 (0.7%) | 12 (2.0%) |
| Missing | 198 | 197 | 1 |
| **If ˂ 1 day** | **N=1599** | **N=1174** | **N=425** |
| ˂ 1 h + 1-8 hours | 1,397 (87%) | 1,002 (85%) | 395 (92%) |
| 9-16 hours | 176 (11%) | 148 (13%) | 28 (6.6%) |
| 17-24 hours | 26 (2%) | 24 (2.0%) | 2 (6.6%) |
