# Supplementary material for: Demographics, Causes, and Outcome of Traumatic Brain Injury among Trauma Cases in Cameroon: A Multi-Center Five Year's Retrospective Study
Source: Neurotrauma Rep. 2022 Dec 26;3(1):569–83. doi: 10.1089/neur.2022.0053 (PMC9879018; doi:10.1089/neur.2022.0053)
Supplement: Supplemental data [file Supp_TableS3.docx]

**Supplementary table III:** Causes of TBI according to age, gender, professional groups: Assaults

| **Characteristic** | **Other cause N = 5,418^1^** | **Assaults N = 830^1^** | **p-value** |
| --- | --- | --- | --- |
| **Age** |  |  | ˂0.001 |
| ˂15 | 398 (7.3%) | 15 (1.8%) |  |
| 15-45 | 3,955 (73%) | 734 (89%) |  |
| 46-60 | 628 (12%) | 63 (7.6%) |  |
| ˃60 | 437 (8.1%) | 18 (2.2%) |  |
| **Gender** |  |  | 0.5 |
| Female | 1,077 (20%) | 173 (21%) |  |
| Male | 4,341 (80%) | 657 (79%) |  |
| **Profession** |  |  | ˂0.001 |
| Bike rider | 999 (18%) | 42 (5.1%) |  |
| Construction workers | 318 (5.9%) | 33 (4.0%) |  |
| Drivers | 172 (3.2%) | 14 (1.7%) |  |
| Employment in service | 768 (14%) | 109 (13%) |  |
| Health personnel | 12 (0.2%) | 0 (0%) |  |
| Infants | 89 (1.6%) | 2 (0.2%) |  |
| Manual workers | 497 (9.2%) | 93 (11%) |  |
| Security | 81 (1.5%) | 34 (4.1%) |  |
