# Supplementary material for: Demographics, Causes, and Outcome of Traumatic Brain Injury among Trauma Cases in Cameroon: A Multi-Center Five Year's Retrospective Study
Source: Neurotrauma Rep. 2022 Dec 26;3(1):569–83. doi: 10.1089/neur.2022.0053 (PMC9879018; doi:10.1089/neur.2022.0053)
Supplement: Supplemental data [file Supp_TableS2.docx]

**Supplementary table II:** Causes of TBI according to age, gender and professional groups: Falls

| **Characteristic** | **Other cause** **N = 5,667^1^** | **Falls N = 581^1^** | **P-value** |
| --- | --- | --- | --- |
| **Age** |  |  | ˂0.001 |
| ˂15 | 236 (4.2%) | 177 (30%) |  |
| 15-45 | 4,444 (78%) | 245 (42%) |  |
| 46-60 | 632 (11%) | 59 (10%) |  |
| ˃ 60 | 355 (6.3%) | 100 (17%) |  |
| **Gender** |  |  | ˂0.001 |
| Female | 1,064 (19%) | 186 (32%) |  |
| Male | 4,603 (81%) | 395 (68%) |  |
| **Profession** |  |  | ˂0.001 |
| Bike rider | 1,038 (18%) | 3 (0.5%) |  |
| Construction workers | 298 (5.3%) | 53 (9.1%) |  |
| Drivers | 185 (3.3%) | 1 (0.2%) |  |
| Employment in service | 809 (14%) | 68 (12%) |  |
| Health personnel | 11 (0.2%) | 1 (0.2%) |  |
| Infants | 27 (0.5%) | 64 (11%) |  |
| Manual workers | 553 (9.8%) | 37 (6.4%) |  |
| Security | 108 (1.9%) | 7 (1.2%) |  |
| Students | 869 (15%) | 156 (27%) |  |
| Technicians | 103 (1.8%) | 7 (1.2%) |  |
| Traders | 681 (12%) | 25 (4.3%) |  |
| Unemployed | 897 (16%) | 140 (24%) |  |
| Other | 88 (1.6%) | 19 (3.3%) |  |
